# ZC3H13‐mediated m6A stabilization of CCND1 promotes malignant progression and is associated with poor anti‐PD‐1 response in HNSCC

**DOI:** 10.1002/ctm2.70750

**Published:** 2026-07-27

**Authors:** Wenqing Chen, Yun Li, Shuang Chen, Caihua Zhang, Zhi Liu, Kai Sun, Yingyi Li, Chunlong Yang, Wenbin Lei, Kang Li

**Affiliations:** ^1^ Otorhinolaryngology Hospital The First Affiliated Hospital Sun Yat‐sen University Guangzhou Guangdong China; ^2^ Department of Laboratory Medicine and Center for Translational Medicine The First Affiliated Hospital Sun Yat‐sen University Guangzhou China; ^3^ Sun Yat‐sen University Cancer Center Guangzhou China; ^4^ Clinical Research Center Affiliated Hospital of Guangdong Medical University Zhanjiang Guangdong China

**Keywords:** CCND1, immunotherapy resistance, m6A modification, ZC3H13

## Abstract

**Background:**

Resistance to immune checkpoint blockade substantially limits its clinical efficacy in head and neck squamous cell carcinoma(HNSCC). ZC3H13 is a component of the N6‐methyladenosine writer complex, but its roles in HNSCC progression and response to anti‐programmed cell death protein 1(anti‐PD‐1) therapy remain unclear.

**Methods:**

The expression and clinical relevance of ZC3H13 were evaluated using clinical cohorts and publicly available transcriptomic datasets. Gain‐ and loss‐of‐function experiments were performed to determine the effects of ZC3H13 on the malignant phenotypes of HNSCC cells. An epithelial‐specific ZC3H13 conditional knockout mouse model of 4‐nitroquinoline‐1‐oxide‐induced oral tumorigenesis was used to assess tumor development and responsiveness to anti‐PD‐1 therapy. N6‐methyladenosine modification, RNA stability and functional rescue assays were conducted to investigate the underlying molecular mechanism.

**Results:**

ZC3H13 was upregulated in HNSCC and was associated with poor prognosis and a limited response to anti‐PD‐1 treatment. ZC3H13 promoted the proliferation and invasion of HNSCC cells, whereas epithelial‐specific ablation of ZC3H13 suppressed oral tumorigenesis and enhanced the therapeutic efficacy of anti‐PD‐1 treatment. Mechanistically, ZC3H13 regulated the N6‐methyladenosine modification of cyclin D1(CCND1) mRNA and promoted its IGF2BP1‐dependent stabilization, thereby contributing to malignant tumor phenotypes and alterations in immunosuppressvie features.

**Conclusions:**

The ZC3H13/IGF2BP1/CCND1 regulatory axis contributes to HNSCC progression and resistance to anti‐PD‐1 therapy. These findings identify ZC3H13 as a potential therapeutic target for improving the efficacy of anti‐PD‐1 treatment in HNSCC.

## Key points

- ZC3H13 is upregulated in HNSCC and is associated with poor prognosis and reduced response to anti‐PD‐1 therapy.
- ZC3H13 promotes HNSCC progression by enhancing the m6A‐dependent, IGF2BP1‐mediated stabilization of CCND1 mRNA.
- Epithelial‐specific ablation of ZC3H13 suppresses oral tumorigenesis and improves the response to PD‐1 blockade, identifying ZC3H13 as a potential therapeutic target.

## Graphical abstract

ZC3H13 promotes HNSCC progression and anti‐PD‐1 resistance by enhancing IGF2BP1‐mediated stabilization of CCND1 mRNA in an m6A‐dependent manner. Targeting ZC3H13 suppresses oral tumorigenesis and improves response to PD‐1 blockade.

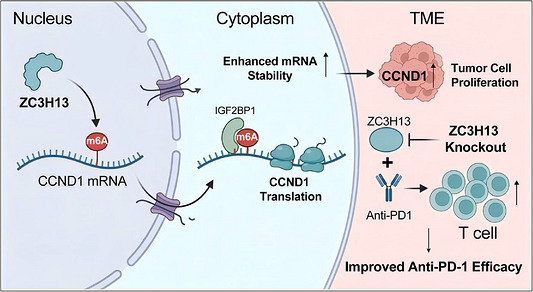

## 1 INTRODUCTION

The head and neck squamous cell carcinoma (HNSCC) remains a major public health challenge with feature of pronounced molecular heterogeneity and a tumour microenvironment (TME) with a prominently immunosuppressive landscape.^1^, ^2^, ^3^, ^4^ While immune checkpoint blockade (ICB) blocking the programmed cell death protein 1/programmed death‐ligand 1 (PD‐1/PD‐L1) axis has established a new standard of care, recent clinical follow‐up studies indicate that sustained therapeutic efficacy is achieved in only a subset of patients, primarily due to the emergence of acquired resistance.^5^, ^6^, ^7^ Longitudinal single‐cell RNA sequencing (scRNA‐seq) profiling has recently uncovered specific malignant cell states that evolve under the selective pressure of PD‐1 inhibition, actively remodelling the TME to foster an immune‐cold niche characterized by the terminal exhaustion of effector T cells.^8^, ^9^, ^10^, ^11^

N6‐methyladenosine (m6A), a typical prevalent internal modification of eukaryotic mRNA, serves as critical epitranscriptomic layer regulating the plasticity of drug‐resistant clones.^12^, ^13^ The deposition of m6A is orchestrated by the m6A methyltransferase complex (MTC), in which ZC3H13 serves as a pivotal scaffold protein essential for the nuclear localization and enzymatic processivity of the complex.^14^, ^15^ While the role of ZC3H13 has been historically debated, contemporary studies have increasingly linked its dysregulation to the metabolic and epigenetic reprogramming of solid tumors.^16^, ^17^ However, the specific contribution of ZC3H13‐mediated m6A modification to the acquired resistance of HNSCC and its subsequent influence on the immune microenvironment remains poorly defined.

By integrating multi‐omics profiles of HNSCC patients undergoing anti‐PD‐1 therapy, we identified a ZC3H13‐driven malignant subpopulation that is significantly enriched in post‐treatment lesions. Our molecular dissection identified Cyclin D1 (CCND1) as the primary downstream functional target of this axis. CCND1 is essential for the G1/S cell‐cycle transition, and its overexpression remains a major driver of HNSCC progression and poor clinical prognosis.^18^, ^19^ Beyond its cell cycle functions, recent evidence has highlighted that the CCND1/CDK4/6 axis facilitates immune evasion by modulating the release of cytokines and impairing the antigen presentation machinery.^20^, ^21^ Furthermore, recent studies on m6A readers have refined the role of the IGF2BP family (IGF2BP1/2/3) as crucial stability readers that protect oncogenic transcripts from ribonuclease‐mediated decay.^22^, ^23^ This suggests that ZC3H13 may link intrinsic tumour growth to immune‐suppressive features through the m6A‐IGF2BP1‐mediated stabilization of CCND1 mRNA.

Here, we examined the contribution of the ZC3H13/CCND1 axis in HNSCC malignant progression and poor response to anti‐PD‐1 treatment. We demonstrate that ZC3H13 promotes m6A modification of CCND1 mRNA and enhances its IGF2BP1‐associated transcript stability. This process appears to accelerate intrinsic proliferation and may be linked to immune‐suppressive microenvironmental features, including exhaustion‐related transcriptional changes in CD4+ T cells.^24^ By combining clinical validation, methylated RNA immunoprecipitation sequencing (MeRIP‐seq), and epidermis‐specific conditional knockout (cKO) models, we nominate the ZC3H13/IGF2BP1/CCND1 axis as a candidate regulator associated with HNSCC progression and poor anti‐PD‐1 response.

## 2 RESULTS

### 2.1 Single‐cell transcriptomic landscape reveals a post‐treatment‐enriched ZC3H13‐high malignant subpopulation and its clinical prognostic value

**FIGURE 1:**
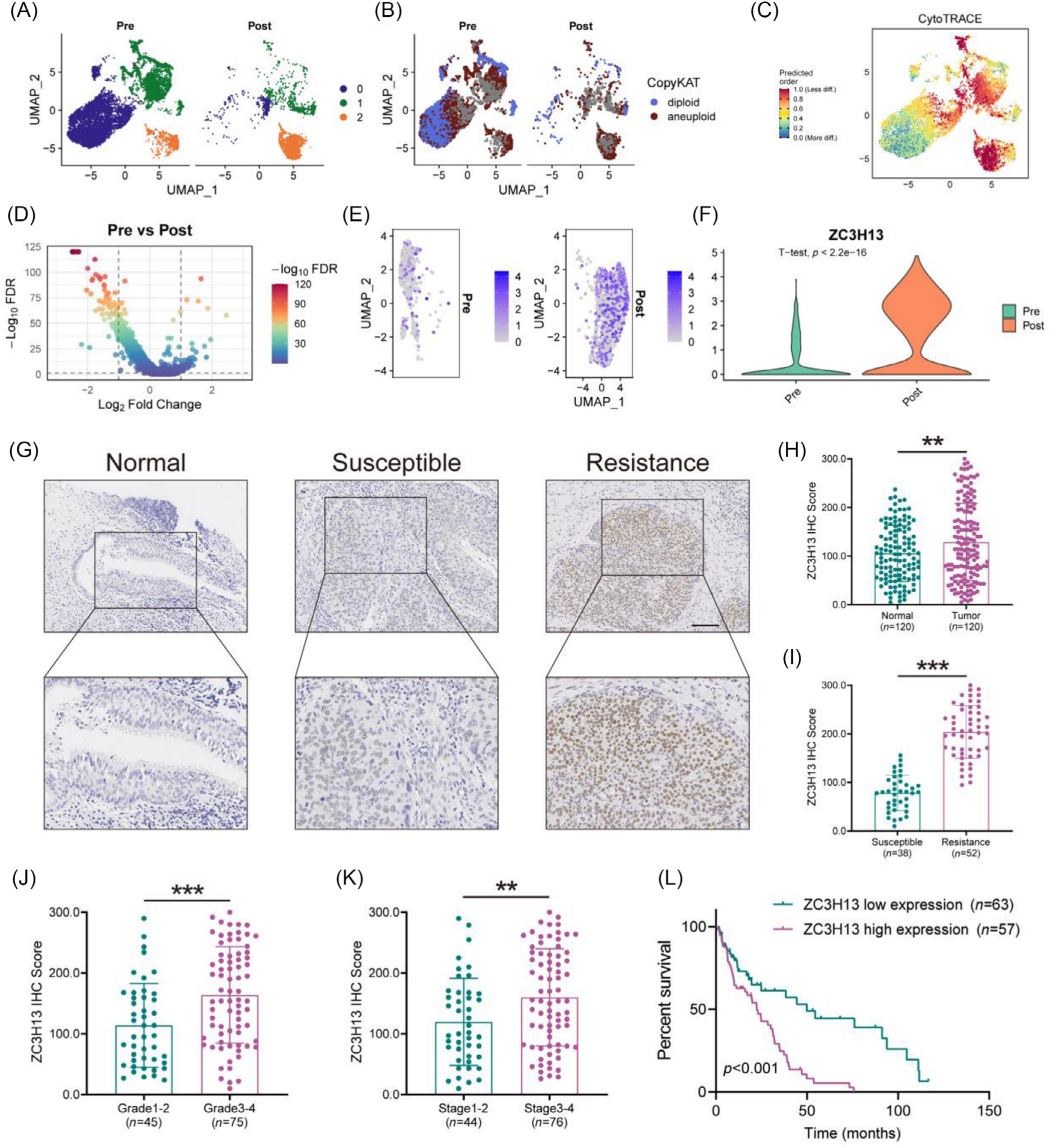
Characterization of ZC3H13 expression in HNSCC and its predictive value for immunotherapy response. (A) UMAP visualization illustrating the distribution of tumour cell populations in HNSCC samples before (Pre) and after (Post) immunotherapy. (B) UMAP plots identifying aneuploid (malignant) and diploid (non‐malignant) cells based on genomic copy number variations inferred by the CopyKAT algorithm. (C) CytoTRACE analysis ranking the differentiation potential of malignant cells; higher scores indicate a lower differentiation state and higher stemness potential. (D) Volcano plot displaying differentially expressed genes (DEGs) in malignant cells between the Pre and Post groups. (E) UMAP feature plots exhibiting the single‐cell expression levels of ZC3H13 in malignant cells from Pre and Post immunotherapy groups. (F) Violin plots quantifying *ZC3H13* transcript levels in malignant cells before and after treatment. (G) Representative immunohistochemistry (IHC) images demonstrating ZC3H13 expression in normal oral tissues, anti‐PD‐1 responder tissues, and anti‐PD‐1 non‐responder tissues. Scale bar, 100µm. (H) Statistical comparison of ZC3H13 IHC scores between normal and tumour tissues in a clinical cohort of 120 paired samples. (I) Differential analysis of ZC3H13 protein expression between immunotherapy‐susceptible (*n* = 38) and resistance (*n* = 52) patients. (J) Comparison of ZC3H13 IHC scores stratified by histological grade (Grade 1–2 vs. Grade 3–4). (K) Comparison of ZC3H13 IHC scores stratified by clinical stage (Stage 1–2 vs, Stage 3–4). (L) Kaplan–Meier survival curves showing the difference in overall survival (OS) between ZC3H13 high‐expression and low‐expression groups. Patients were dichotomized according to the median H‐score of the corresponding marker. (M) Forest plot showing multivariate Cox proportional hazards regression analysis of overall survival in the HNSCC clinical cohort. Variables included ZC3H13 expression status, age, sex, histological grade and TNM stage. Hazard ratios (HRs), 95% confidence intervals (CIs), and *p* values are shown. Public scRNA‐seq data used in panels A–F were obtained from GEO accession GSE195832; public bulk RNA‐seq validation data were obtained from TCGA‐HNSC through the GDC Data Portal.

To elucidate the dynamic evolution of the TME in HNSCC during anti‐PD‐1 treatment, we analysed related scRNA‐seq data from paired pre‐treatment (Pre) and post‐treatment (Post) samples. Cell type annotation defined the major lineages and their compositional shifts before and after treatment (Figure S1A). Unsupervised clustering of the tumour cell compartment identified three distinct subclusters (Clusters 0, 1 and 2) (Figure 1A, S1B). Notably, Cluster 2 was significantly enriched in the post‐treatment samples (Figure 1E, Figure S1C), suggesting that this cell population represents a post‐treatment‐enriched malignant state. Because direct lineage tracing was not available in this public dataset, we avoided defining this cluster as an acquired drug‐resistant clone. Copy number karyotyping of aneuploid tumours (CopyKAT) analysis confirmed the aneuploid nature of these cells, verifying their malignant identity (Figure 1B).

Further phenotypic analysis revealed the aggressive nature of this post‐treatment‐enriched malignant subpopulation. CytoTRACE analysis evaluated the distinct differentiation states among the clusters (Figure 1C). Functional scoring showed that Cluster 2 exhibited higher Epithelial‐Mesenchymal Transition (EMT) and Cell Cycle scores (Figure S1D), indicating a highly proliferative and invasive phenotype. Differential gene expression analysis between pre‐ and post‐treatment tumour cells (Figure 1D) demonstrated that the ZC3H13 gene was most significantly upregulated in the post‐treatment‐enriched malignant population (*p* < 2.2e‐16) (Figure 1F). These transcriptomic results indicate that ZC3H13 is a molecular feature of the post‐treatment‐enriched malignant cluster.

To further confirm the scRNA‐seq findings at the protein level and within larger clinical cohorts, we assessed ZC3H13 protein expression levels using immunohistochemistry (IHC) on tissue microarrays. The results indicated that tumour samples exhibited markedly higher ZC3H13 abundance than adjacent normal tissues (Figure 1G,H). In an independent cohort of patients who received anti‐PD‐1‐based immunotherapy, ZC3H13 expression was significantly elevated in tumours from anti‐PD‐1 non‐responders relative to those from anti‐PD‐1 responders (Figure 1G,I), supporting an association between high ZC3H13 level and unfavourable response to anti‐PD‐1 therapy.

Furthermore, clinicopathological correlation analysis showed that elevated ZC3H13 expression was enriched in tumours with higher pathological grades (Grade 3–4) (Figure 1J) and advanced clinical stage (Stage 3–4) (Figure 1K). Kaplan–Meier curves revealed markedly reduced overall survival (OS) among patients with high ZC3H13 expression (*p* < .001) (Figure 1L). To further determine whether ZC3H13 expression was independently associated with patient prognosis, we performed multivariate Cox proportional hazards regression analyses. Importantly, after adjustment for age, sex, histological grade, and tumour‐node‐metastasis (TNM) stage, high ZC3H13 expression independently predicted shorter OS (hazard ratio (HR) = 1.96, 95% confidence interval (CI): 1.14–3.39, *p* = .015; Figure 1M). These validation results are consistent with Bulk RNA‐seq analysis from public databases, which also confirmed the significant upregulation of ZC3H13 transcripts in tumour tissues and high pathological grades (Figure S1E,S1F). Collectively, these findings indicate that ZC3H13‐high malignant cells are preferentially enriched following anti‐PD‐1 treatment, and that elevated ZC3H13 expression is linked to unfavourable prognosis and reduced therapeutic responsiveness in HNSCC.

### 2.2 ZC3H13 promotes the proliferation, migration and invasion of HNSCC cells in vitro

**FIGURE 2:**
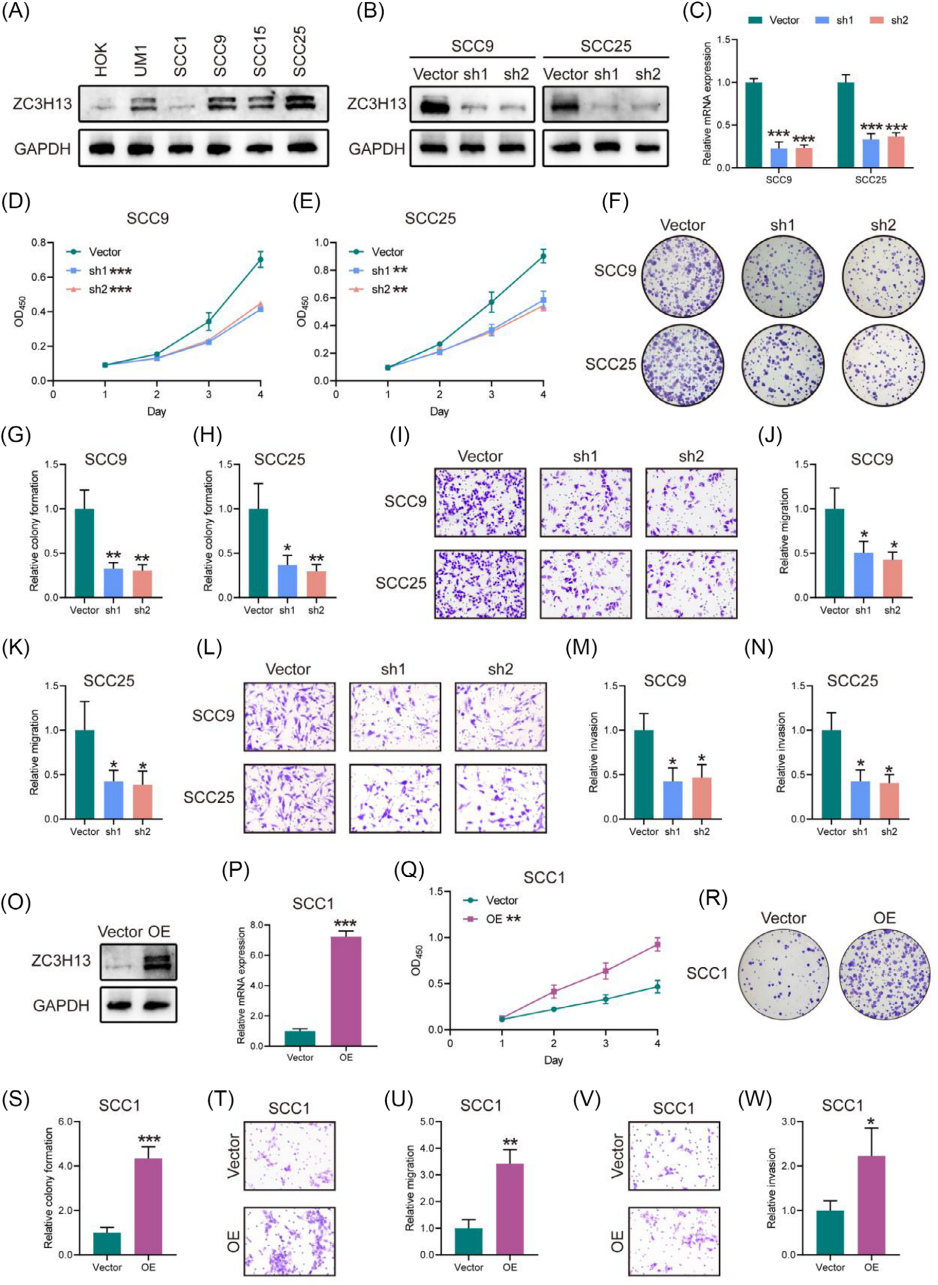
ZC3H13 promotes the proliferation, migration and invasion of HNSCC cells. (A) Basal protein expression levels of ZC3H13 in normal human oral keratinocytes (HOK) and various HNSCC cell lines (UM1, SCC1, SCC9, SCC15, SCC25) were detected by Western blotting. (B and C) The knockdown efficiency of ZC3H13 in SCC9 and SCC25 cells transfected with shRNAs (sh1, sh2) was confirmed by Western blotting (B) and RT‐qPCR (C). (D and E) Cell proliferation kinetics of SCC9 and SCC25 cells following ZC3H13 knockdown were assessed via CCK‐8 assays. (F–H) Colony formation assays and corresponding quantitative analyses were performed to evaluate the long‐term proliferative capacities of SCC9 and SCC25 cells after ZC3H13 depletion. (I‐K) Transwell migration assays and quantification demonstrating the effect of ZC3H13 knockdown on the motility of SCC9 and SCC25 cells. (L‐N) Transwell invasion assays revealing the invasive abilities of SCC9 and SCC25 cells through Matrigel following ZC3H13 knockdown. (O and P) The overexpression (OE) efficiency of ZC3H13 in SCC1 cells was verified by Western blotting (O) and RT‐qPCR (P). (Q) CCK‐8 assay evaluating the proliferation of SCC1 cells following ZC3H13 overexpression. (R and S) Colony formation assay and quantitative analysis of SCC1 cells with ZC3H13 overexpression. (T–W) Transwell assays evaluating the migration (T and U) and invasion (V and W) capacities of SCC1 cells after ZC3H13 overexpression. Data are presented as the mean ± standard deviation (SD) from three independent experiments. *p < .05, **p < .01, ***p < .001.

To explore the involvement of ZC3H13 in HNSCC, we first examined endogenous ZC3H13 protein expression in human oral keratinocytes (HOKs) and multiple HNSCC cell lines using Western blot (Figure 2A). The results indicated that most HNSCC cell lines displayed increased ZC3H13 protein expression compared with HOK cells. Based on these expression profiles, we selected SCC9 and SCC25 cells, which exhibited relatively high baseline expression, for subsequent knockdown (KD) experiments, and SCC1 cells, which showed low expression, for overexpression (OE) experiments. We successfully established stable KD and OE cell models using short hairpin RNAs (shRNAs) targeting ZC3H13 (sh1, sh2) and an overexpression plasmid, respectively. The transfection efficiencies were robustly validated at the protein (Figure 2B,O) and mRNA (Figure 2C,P) levels.

Subsequently, we determined how ZC3H13 influences the growth of HNSCC cell. Cell Counting Kit‐8 (CCK‐8) analysis revealed markedly reduced proliferative capacity in SCC9 and SCC25 cells after ZC3H13 knockdown (Figure 2D,E). Colony formation assays demonstrated markedly reduced colony‐forming ability in both ZC3H13‐silenced cell lines (Figure 2F–H). Conversely, ectopic overexpression of ZC3H13 in SCC1 cells markedly accelerated cell proliferation (Figure 2Q) and increased the number of formed colonies (Figure 2R,S).

Given that the scRNA‐seq analysis in Figure 1 suggested a highly active EMT signature in the post‐treatment‐enriched malignant subpopulation, we further performed Transwell assays to evaluate the specific role of ZC3H13 in metastatic potential. The data indicated that ZC3H13 knockdown markedly impaired the capability of SCC9 and SCC25 cells to traverse the membrane and invade Matrigel, indicating impaired migration (Figure 2I–K) and invasion (Figure 2L–N) capacities. In contrast, overexpression of ZC3H13 robustly enhanced the in vitro migration (Figure 2T,U) and invasion potential (Figure 2V,W) of SCC1 cells. These in vitro functional data indicate that ZC3H13 acts as an oncogenic driver of HNSCC malignant progression, which is highly consistent with its clinical correlation with acquired resistance and poor patient prognosis.

### 2.3 Epithelial ZC3H13 deficiency inhibits 4NQO‐induced HNSCC progression and enhances anti‐PD‐1 responsiveness in vivo

**FIGURE 3:**
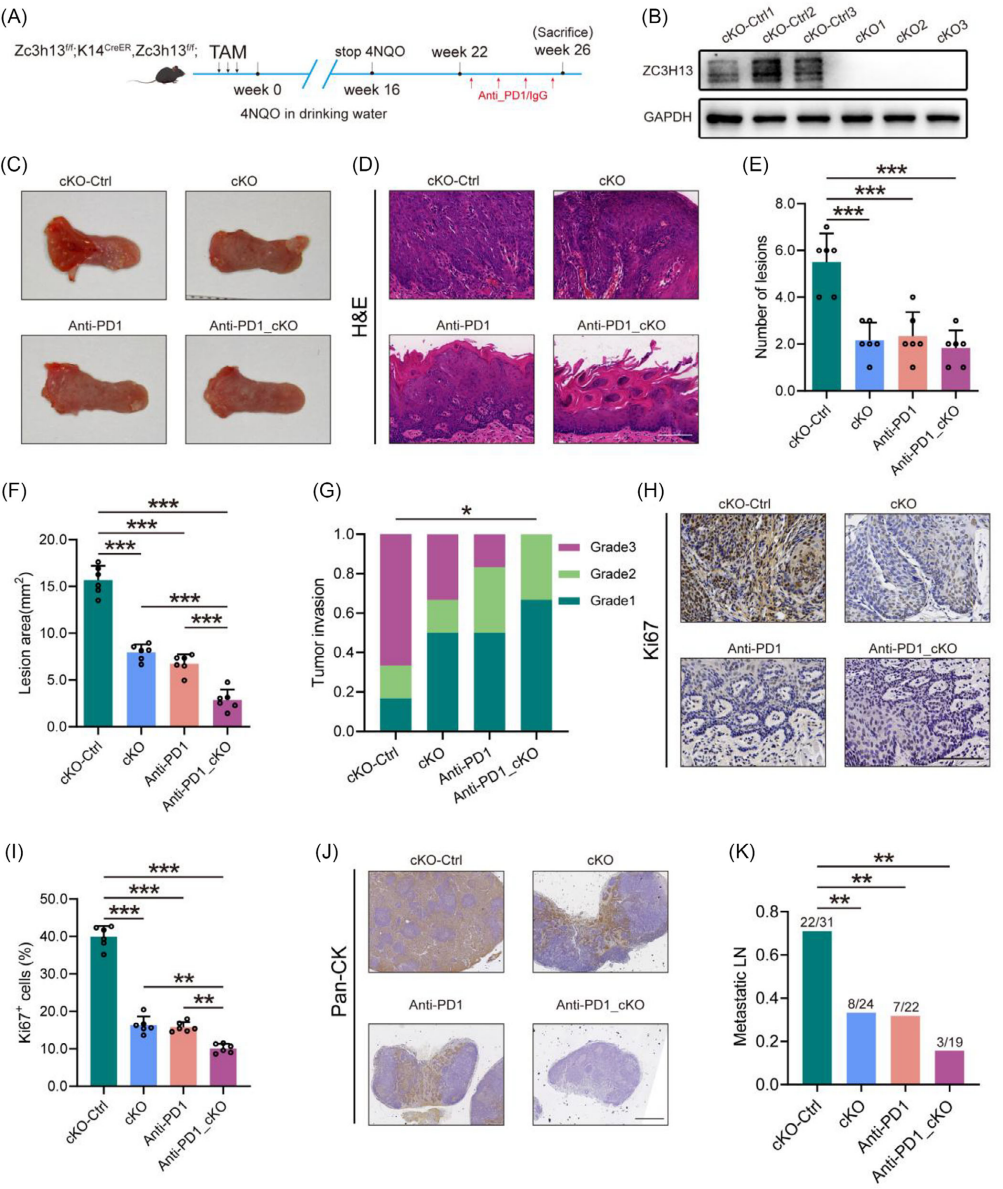
ZC3H13 deficiency enhances the antitumor effect of anti‐PD‐1 therapy in the 4NQO‐induced HNSCC model. (A) Schematic diagram of the 4NQO‐induced HNSCC transgenic mouse model and the anti‐PD‐1 treatment timeline. Tamoxifen (TAM) was utilized to induce epithelial‐specific ZC3H13 conditional knockout (cKO) in *K14^CreER^*; *ZC3H13^fl/fl^* mice. (B) Western blot analysis confirming the in vivo knockout efficiency of ZC3H13 in tissues from control (cKO‐Ctrl) and conditional knockout (cKO) mice. (C) Representative gross morphological images of tongues from mice in the cKO‐Ctrl, cKO, Anti‐PD1 and Anti‐PD1_cKO groups. (D) Representative H&E staining images of tongue lesions from the indicated groups. Scale bar, 100µm. (E and F) Quantification of the number of lesions (E) and lesion area (F) in the tongues of mice across the different experimental groups. (G) Proportions of tumour invasion histological grades (Grade 1, 2 and 3) among the different groups. (H and I) Representative immunohistochemistry (IHC) images (H) and quantitative analysis (I) of the proportion of cells positive for the proliferation marker Ki67 in tongue tumour tissues. Scale bar, 100µm. (J and K) Representative Pan‐CK IHC images (J) of lymph nodes and quantitative statistics (K) evaluating the rate of lymph node metastasis (Metastatic LN) across the groups. Scale bar, 500µm. Data are presented as the mean ± SD. *p < .05, **p < .01, ***p < .001. The number of mice analysed is indicated in the figure. One‐way ANOVA or Fisher's exact test was used as appropriate.

To evaluate the oncogenic role of ZC3H13 and its impact on immunotherapy within an immunocompetent in vivo environment, we generated a keratin 14 (K14) promoter‐driven, epidermis‐specific ZC3H13 conditional knockout (cKO) mouse model (Zc3h13fl/fl; K14CreER). Using tamoxifen (TAM) induction followed by the classical 4‐nitroquinoline‐1‐oxide (4NQO) drinking water protocol, we established a primary HNSCC model and introduced anti‐PD‐1 treatment at the advanced‐stage disease (Figure 3A). The effective ablation of ZC3H13 protein in the tongue tissues following TAM treatment was confirmed by Western blot (Figure 3B).

Macroscopic observation and pathological quantification revealed that, compared to the large and dense tumour masses on the tongues of the control group (cKO‐Ctrl), either ZC3H13 deletion (cKO) alone or anti‐PD‐1 monotherapy significantly reduced the number of tongue lesions (Figure 3C,E) and decreased the tumour lesion area (Figure 3F). More importantly, the combination of ZC3H13 knockout and anti‐PD‐1 treatment (Anti‐PD1_cKO) exhibited the most profound tumour‐suppressive effect, minimizing the tumour burden to the lowest level. These findings suggest that epithelial ZC3H13 deficiency enhances anti‐PD‐1 responsiveness in this immunocompetent 4NQO‐induced HNSCC model.

Further histological analyses reinforced these macroscopic findings. The result of haematoxylin and eosin (H&E) staining and tumour invasion grading demonstrated a high proportion of deeply invasive carcinomas (Grade 3) in the cKO‐Ctrl group, whereas single cKO or anti‐PD‐1 treatment attenuated the invasion severity. In the combination therapy group, the vast majority of lesions were restricted to the early stage (Grade 1), displaying the least malignant histological features (Figure 3D,G). Consistently, Ki67 IHC staining revealed the greatest reduction in tumour cell proliferative activity in the combination treatment group (Figure 3H,I). Finally, we evaluated lymph node metastasis by pan‐cytokeratin (Pan‐CK) staining of cervical lymph nodes. The results showed a remarkably high metastatic rate in the cKO‐Ctrl group (22/31), whereas the loss of ZC3H13 significantly reduced the incidence of metastasis (8/24), which was further restrained upon combination with anti‐PD‐1 therapy (3/19) (Figure 3J,K). Collectively, these in vivo data suggest a role for ZC3H13 in promoting HNSCC malignant progression and modulating responsiveness to anti‐PD‐1 therapy.

### 2.4 ZC3H13 regulates cell cycle pathways by mediating m6A modification on *CCND1* mRNA

**FIGURE 4:**
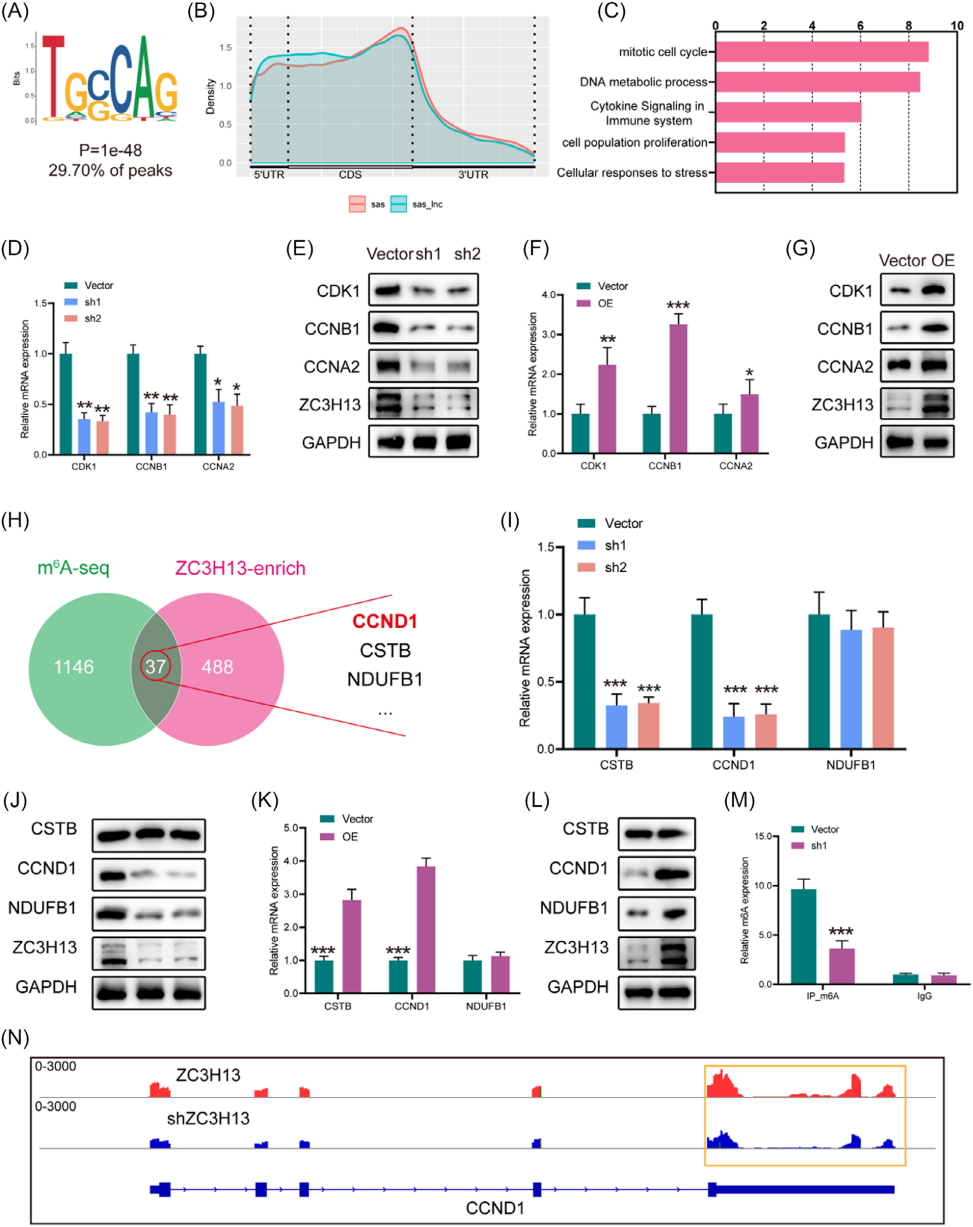
Identification of CCND1 as a downstream transcript of ZC3H13 via MeRIP‐seq and transcriptomic analyses. (A) The most highly enriched m6A consensus motif identified from MeRIP‐seq data. (B) Density plot showing the distribution of identified m6A peaks across different regions of mRNA transcripts, including the 5′ untranslated region (5′ UTR), coding sequence (CDS), and 3′ untranslated region (3′ UTR). (C) Gene Ontology (GO) biological process enrichment analysis of genes with ZC3H13‐regulated m6A changes. (D and E) The mRNA and protein expression levels of cell cycle‐related genes (CDK1, CCNB1, CCNA2) following ZC3H13 knockdown (sh1, sh2) were assessed by RT‐qPCR (D) and Western blotting (E). (F and G) The expression changes of the aforementioned cell cycle‐related genes after ZC3H13 overexpression (OE) were determined by RT‐qPCR (F) and Western blotting (G). (G) Venn diagram showing the overlap between MeRIP‐seq‐identified candidate m6A‐regulated transcripts and genes enriched in the ZC3H13‐high post‐treatment malignant cell population, identifying 37 potential downstream candidates, including CCND1, CSTB, and NDUFB1. (I‐J) RT‐qPCR (I) and Western blot (J) validation of the suppressive effect of ZC3H13 knockdown on the expression of candidate target genes (CSTB, CCND1, NDUFB1). (K‐L) RT‐qPCR (K) and Western blot (L) validation demonstrating the promoting effect of ZC3H13 overexpression on target gene expression. (M) MeRIP‐qPCR assay quantifying the changes in m6A modification abundance on *CCND1* mRNA following ZC3H13 depletion. (N) Integrative Genomics Viewer (IGV) browser tracks displaying the distribution and abundance of sequencing peaks along the CCND1 transcript in the control (ZC3H13) and knockdown (shZC3H13) groups. Data are presented as mean ± SD from three independent biological experiments unless otherwise indicated. *p < .05, **p < .01, ***p < .00.

To define the molecular basis of ZC3H13‐mediated HNSCC progression, transcriptome‐wide m6A sequencing (m6A‐seq) was performed. Motif enrichment analysis identified the highly conserved consensus m6A motif sequence “TGCCAG” (Figure 4A). Peak distribution analysis revealed that m6A modification sites were obviously enriched in the coding sequences (CDS) and near the 3′ untranslated region (3′ UTR) of mRNAs (Figure 4B), which closely aligns with the known topological distribution of $m^6A$ modifications. The enrichment analyses (Gene Ontology (GO)/ Kyoto Encyclopedia of Genes and Genomes (KEGG)) of the differentially modified genes showed an obvious enrichment in pathways such as ‘mitotic cell cycle’ and ‘cell population proliferation’ (Figure 4C). To validate this, we examined classic cell cycle regulatory proteins. These results indicated that ZC3H13 knockdown markedly downregulated CDK1, CCNB1 and CCNA2 expression (Figure 4D,E), whereas ZC3H13 overexpression increased their expression (Figure 4F,G).

To further pinpoint the direct and clinically relevant target genes of ZC3H13, we intersected the potential targets identified by m6A‐seq with the signature gene set of the ZC3H13‐high post‐treatment‐enriched malignant subpopulation derived from our aforementioned scRNA‐seq analysis (ZC3H13‐enriched signature genes) using a Venn diagram. This intersection yielded 37 candidate genes, including *CCND1*, a central mediator of cell cycle, as well as *CSTB* and *NDUFB1* (Figure 4H). Subsequent quantitative real‐time PCR (RT‐qPCR) and Western blot analyses confirmed that ZC3H13 knockdown substantially reduced CCND1 and CSTB levels at both the transcript and protein levels (Figure 4I,J), while ZC3H13 overexpression significantly upregulated their expression (Figure 4K,L). Among these candidates, CCND1 was prioritized for further investigation because it was identified as a ZC3H13‐regulated m6A‐modified transcript and was consistently regulated by ZC3H13 at both the mRNA and protein levels. Moreover, CCND1 encodes Cyclin D1, a central regulator of G1/S cell progression with established relevance to HNSCC progression. We therefore focused on CCND1 as a functionally testable downstream effector, while recognizing that other candidate transcripts may also contribute to ZC3H13‐dependent phenotypes. Finally, we confirmed the direct epigenetic regulation of CCND1 mRNA by ZC3H13. Methylated RNA immunoprecipitation quantitative PCR (MeRIP‐qPCR) analysis demonstrated that the specific m6A enrichment level on CCND1 mRNA was significantly reduced upon ZC3H13 depletion (Figure 4M). Furthermore, the Integrative Genomics Viewer (IGV) tracks visually displayed a marked attenuation of m6A sequencing peaks at the 3' terminal region of the CCND1 transcript in ZC3H13‐knockdown cells (Figure 4N, highlighted by the yellow box). Together, these multi‐omics and molecular analyses support CCND1 as a transcript whose m6A modification is regulated by ZC3H13. Because ZC3H13 functions as a scaffold component of the m6A methyltransferase complex rather than a sequence‐specific RNA‐binding protein, our data suggest that ZC3H13 facilitates writer‐complex‐dependent m6A modification of CCND1 mRNA.

### 2.5 ZC3H13‐mediated m6A modification enhances *CCND1* mRNA stability by recruiting IGF2BP1

**FIGURE 5:**
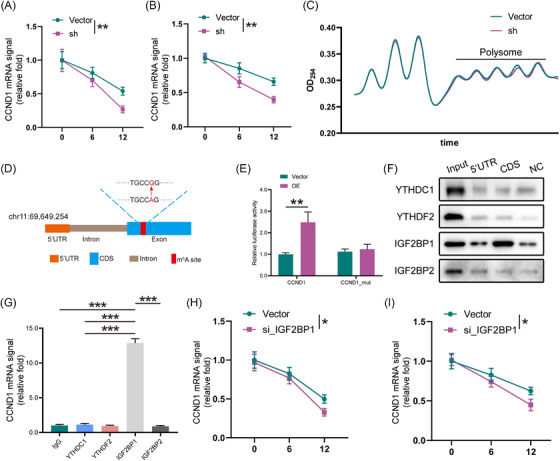
ZC3H13 enhances *CCND1* mRNA stability in an IGF2BP1‐dependent manner. (A and B) Actinomycin D transcription inhibition assay followed by RT‐qPCR to assess the effect of ZC3H13 knockdown (sh) on the degradation rate and stability of *CCND1* mRNA in HNSCC cells. (C) Polysome profiling analysis illustrating the distribution of monosomes and polysomes in control (Vector) and ZC3H13‐depleted cells. (D) Schematic illustration of the predicted m6A modification site on the CCND1 transcript and the construction of wild‐type and mutant dual‐luciferase reporter vectors. The mutant reporter was generated by an A‐to‐G substitution within the predicted m6A consensus motif, changing TGCCAG to TGCCGG. (E) Dual‐luciferase reporter assay evaluating the relative luciferase activity of WT or mutant CCND1 reporters following ZC3H13 overexpression (OE). (F) RNA pulldown assay followed by Western blotting to detect the direct binding of candidate m6A reader proteins (YTHDC1, YTHDF2, IGF2BP1, IGF2BP2) to different regions (5' UTR, CDS) of the CCND1 transcript. (G) RIP‐qPCR assay quantifying the specific enrichment of *CCND1* mRNA by various m6A reader proteins. (H and I) Actinomycin D RNA decay assay showing the effect of IGF2BP1 knockdown on CCND1 mRNA stability. Accelerated CCND1 mRNA degradation after IGF2BP1 depletion supports IGF2BP1 as a reader protein that stabilizes CCND1 mRNA. Data are presented as mean ± SD from three independent biological experiments. Statistical tests were selected according to the experimental design as described in the Statistical Analysis section. *p < .05, **p < .01, ***p < .001.

To determine how ZC3H13‐regulated m6A modification is translated into altered CCND1 expression, we next examined CCND1 mRNA stability and the involvement of candidate m6A reader proteins. RNA decay assays using actinomycin D treatment suggested that ZC3H13 knockdown accelerated the degradation of CCND1 mRNA, resulting in a shortened mRNA half‐life (Figure 5A,B). Polysome profiling showed comparable monosome and polysome distributions between control and ZC3H13‐knockdown cells (Figure 5C), indicating that ZC3H13 depletion did not cause obvious global translational suppression. To further confirm that this regulation is dependent on m6A modification, we introduced an A‐to‐G substitution into the predicted m6A consensus motif, changing TGCCAG to TGCCGG in the CCND1 sequence, and generated wild‐type (WT) and mutant (Mut) dual‐luciferase reporter plasmids (Figure 5D). These results showed that ZC3H13 overexpression markedly enhanced reporter activity driven by the construct containing the WT CCND1 sequence but had no effect on the mutant reporter (Figure 5E). These findings suggest that the predicted m6A site is required, at least in part, for ZC3H13‐mediated regulation of CCND1.

It is well‐established that m6A modifications typically require specific reader proteins to execute their biological functions.^25^, ^26^ To identify the specific reader recognizing the m6A tag on CCND1 mRNA, we performed RNA pull‐down assays. We examined several established m6A reader proteins involved in RNA fate regulation, including YTHDC1, YTHDF2, IGF2BP1 and IGF2BP2. RNA pull‐down and Western blotting result indicated that IGF2BP1 preferentially associated with the CCND1 transcript region containing the predicted m6A‐modified site, whereas YTHDC1, YTHDF2 and IGF2BP2 showed much weaker or non‐specific binding signals under the same conditions (Figure 5F). Subsequent RNA immunoprecipitation quantitative PCR (RIP‐qPCR) assays further corroborated this interaction at the intracellular level, showing that the anti‐IGF2BP1 antibody remarkably enriched the CCND1 transcripts (Figure 5G). IGF2BP1 was silenced to assess its involvement in this regulatory mechanism. The results demonstrated that silencing IGF2BP1 (si_IGF2BP1) phenocopied the effect of ZC3H13 depletion, leading to a significantly accelerated degradation of CCND1 mRNA (Figure 5H,I). Collectively, these findings confirm that ZC3H13‐dependent m6A modification stabilizes CCND1 mRNA by facilitating its association with IGF2BP1.

### 2.6 ZC3H13 promotes HNSCC malignant phenotypes in a CCND1‐dependent manner and positively correlates with CCND1 in clinical tissues

**FIGURE 6:**
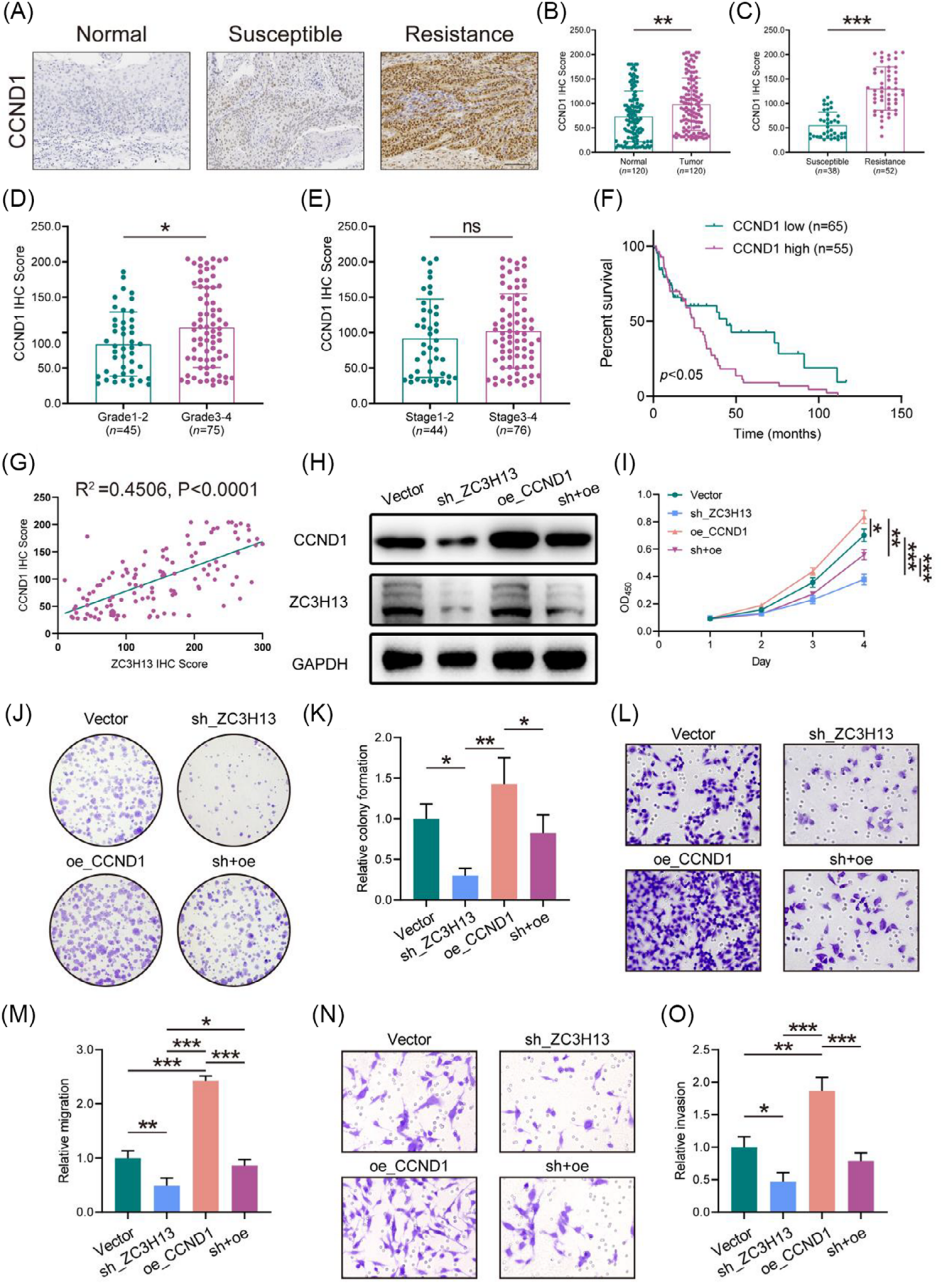
Clinical significance of CCND1 in HNSCC and its rescue effect on ZC3H13‐mediated cellular malignant phenotypes. (A) Representative immunohistochemistry (IHC) images demonstrating CCND1 expression in normal oral tissues, anti‐PD‐1 responder tissues, and anti‐PD‐1 non‐responder tumour tissues. (B) Statistical comparison of CCND1 IHC scores between normal and tumour tissues in a clinical cohort of 120 paired samples. (C) Differential analysis of CCND1 protein expression between anti‐PD‐1 responders (n = 38) and anti‐PD‐1 non‐responders (*n* = 52) patients. (D) Comparison of CCND1 IHC scores stratified by histological grade (Grade 1–2 vs Grade 3–4). (E) Comparison of CCND1 IHC scores stratified by clinical stage (Stage 1–2 vs. Stage 3–4). (F) Kaplan–Meier survival curves showing the difference in overall survival (OS) between CCND1 high‐expression (*n* = 55) and low‐expression (*n* = 65) groups. Patients were dichotomized according to the median H‐score of the corresponding marker. (G) Pearson correlation analysis revealing a significant positive correlation between ZC3H13 and CCND1 protein expression (IHC scores) in HNSCC tumour tissues. (H) Western blot analysis of ZC3H13 and CCND1 protein expression levels in cells co‐transfected with Vector, sh_ZC3H13, oe_CCND1, or sh+oe to validate the efficacy of the rescue experiment. (I) CCK‐8 assay evaluating the rescue effect of CCND1 overexpression on the proliferation inhibition induced by ZC3H13 knockdown in HNSCC cells. (J and K) Colony formation assay and quantitative analysis evaluating the restorative effect of CCND1 on the long‐term proliferative capacity of ZC3H13‐depleted cells. (L and M) Transwell migration assay and quantitative analysis confirming that CCND1 reversed the suppressive effect of ZC3H13 knockdown on cell migration in vitro. (N and O) Transwell invasion assay and quantitative analysis verifying that CCND1 rescued the impaired invasive capability of cells induced by ZC3H13 knockdown. Data are presented as the mean ± SD from three independent experiments. *p < .05, **p < .01, ***p < .001, ns indicates not significant.

To confirm the clinical significance of the target gene CCND1 in HNSCC and its regulatory relationship with ZC3H13, we conducted IHC staining on tissue sections from HNSCC patients. IHC analysis showed that HNSCC tumours displayed substantially higher CCND1 protein abundance than normal controls (Figure 6A,B). Within the patient group receiving anti‐PD‐1‐based immunotherapy, CCND1 expression was higher in anti‐PD‐1 non‐responders than in responders (Figure 6A,C). Further clinicopathological analysis showed that a higher prevalence of elevated CCND1 levels in tumours with advanced pathological grade (Grade 3–4; Figure 6D), but not with clinical stage (Figure 6E). Patients with high CCND1 expression showed shorter OS according to Kaplan–Meier survival analysis (Figure 6F). Moreover, ZC3H13 IHC scores were positively correlated with CCND1 IHC scores in clinical tumour specimens (Figure 6G). These clinical data corroborate the positive regulatory axis between ZC3H13 and CCND1 and suggest that CCND1 also serves as a biomarker associated with poor prognosis and immunotherapy resistance.

To verify whether ZC3H13 drives tumour malignant progression dependently on CCND1, we conducted phenotypic rescue assays. Western blot analysis confirmed that the introduction of a CCND1 overexpression plasmid into ZC3H13‐knockdown cells (sh+oe) effectively restored the downregulation of CCND1 protein caused by ZC3H13 depletion (Figure 6H). Consistently, depletion of ZC3H13 suppressed multiple malignant phenotypes of HNSCC cells, including proliferation (Figure 6I), colony formation (Figure 6J,K), migration (Figure 6L,M), and invasion (Figure 6N,O). Notably, simultaneously overexpressing CCND1 in ZC3H13‐knockdown cells significantly reversed all the aforementioned inhibitory effects. These molecular and cellular data support CCND1 as a key downstream effector through which ZC3H13 promotes HNSCC cell proliferation and metastatic traits.

### 2.7 The post‐treatment tumour microenvironment is enriched in immunosuppressive pathways and CD4^+^ T cell exhaustion features

**FIGURE 7:**
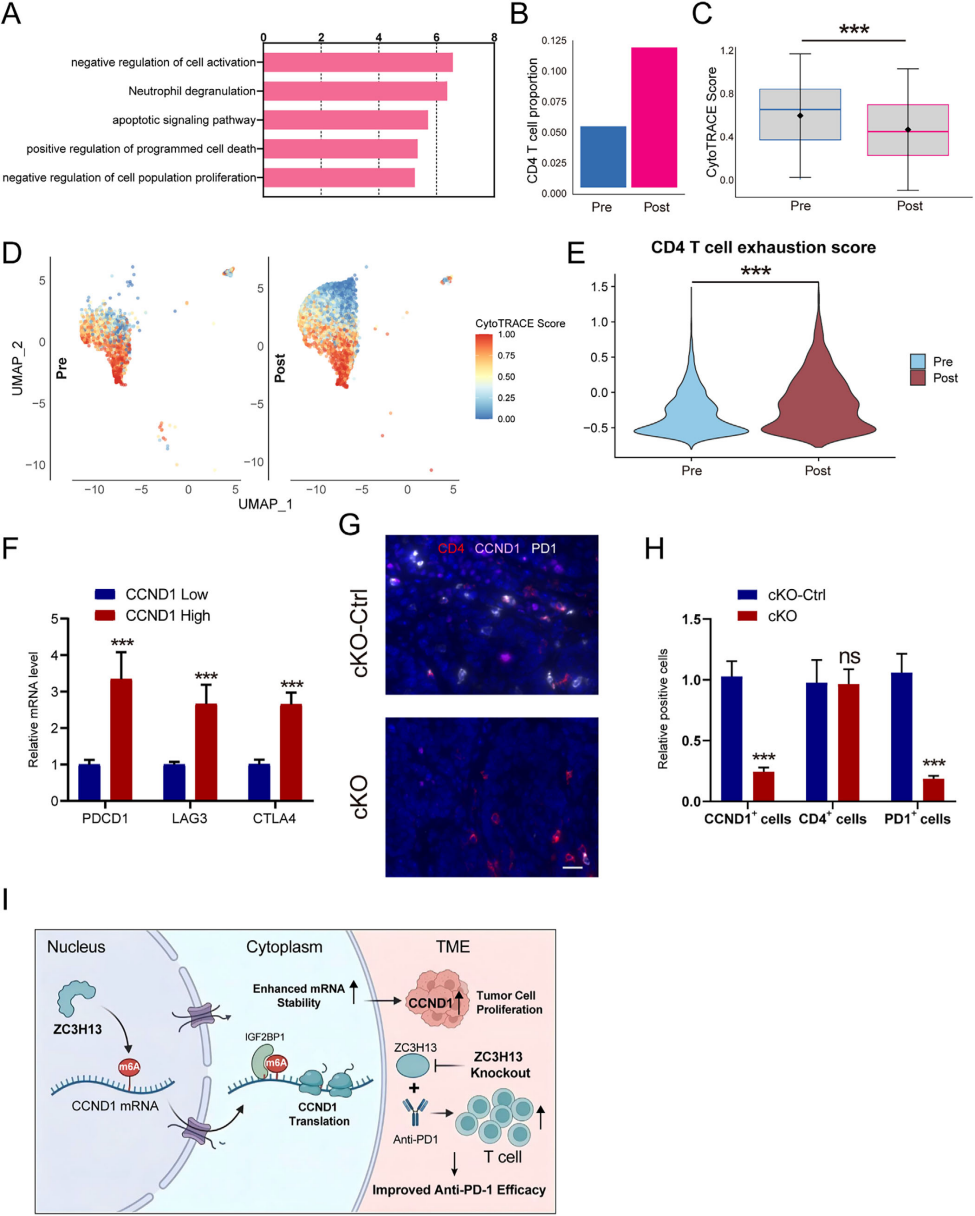
The ZC3H13/CCND1 axis remodels the HNSCC immune microenvironment and the proposed mechanistic model. (A) Gene Ontology (GO) enrichment analysis revealing biological pathways related to the negative regulation of cell activation and apoptosis. (B) Bar plot illustrating the changes in the proportion of CD4^+^ T cells between the Pre‐ and Post‐immunotherapy groups. (C) Boxplot comparing the quantitative CytoTRACE scores before and after immunotherapy (Pre vs. Post). (D) UMAP feature plots displaying the spatial distribution of cell differentiation states (CytoTRACE scores) in Pre‐ and Post‐treatment samples. (E) Violin plot demonstrating the significant difference in CD4+ T cell exhaustion scores between the Pre and Post treatment groups. (F) The relative mRNA expression levels of key immune checkpoint molecules (PDCD1, LAG3, CTLA4) in CCND1‐low and CCND1‐high groups were determined by RT‐qPCR. (G) Representative multiplex immunofluorescence images showing CCND1, CD4, PD‐1 and DAPI staining in mouse HNSCC tissues from control and ZC3H13‐deficient groups. Scale bar, 20 µm. (H) The quantification for the multiplex immunofluorescence (mIF) staining in the tumour microenvironment. (I) Schematic mechanistic model. Data are presented as the mean ± SD. ***p < .001.

To clarify the remodelling effect of anti‐PD‐1 treatment on the TME, we conducted pathway enrichment analysis using the differentially expressed genes (DEGs) between the pre‐treatment (Pre) and post‐treatment (Post) single‐cell transcriptomic data. The results revealed obvious enrichment in pathways like ‘negative regulation of cell activation’, ‘cell population proliferation’, and ‘apoptotic signalling pathway‘ (Figure 7A). These results indicate that the post‐treatment microenvironment exhibits transcriptional features of reduced cellular activation and negative immune regulation.

Subsequently, quantitative analysis of the microenvironmental immune cell populations showed enhanced ratio of CD4+ T cells were more abundant in Post samples than in matched Pre samples (Figure 7B). CytoTRACE analysis showed that post‐treatment CD4^+^ T cells had lower CytoTRACE scores than pre‐treatment CD4^+^ T cells (Figure 7C,D), suggesting reduced transcriptional differentiation potential. We next calculated an exhaustion‐related transcriptional score using a predefined T‐cell exhaustion gene set. Post‐treatment CD4^+^ T cells exhibited higher exhaustion‐related module scores than pre‐treatment CD4^+^ T cells (Figure 7E), suggesting that CD4+ T cells in the post‐treatment TME display exhaustion‐related transcriptional features. To link the intrinsic tumour expression of the target gene CCND1 with these immunosuppressive features, we compared the transcriptional levels of classic T cell exhaustion markers between CCND1‐high and CCND1‐low samples. Consistently, the mRNA levels of the immune checkpoint molecules PDCD1 (PD‐1), lymphocyte activation gene 3 (LAG3), and cytotoxic T‐lymphocyte‐associated protein 4 (CTLA4) were elevated in the CCND1‐high group. (Figure 7F).

To further validate the association between the ZC3H13/CCND1 axis and exhaustion‐associated CD4^+^ T‐cell features at the tissue level, we performed multiplex immunofluorescence staining for CCND1, CD4, PD‐1, and DAPI in mouse HNSCC tissues from the 4NQO‐induced model. Compared with control tumours, ZC3H13‐deficient tumours showed reduced CCND1 expression and a lower abundance of CD4^+^PD‐1^+^ cells within the TME (Figure 7G,H). Quantitative analysis further confirmed a decrease in CCND1 fluorescence intensity and in the fraction of PD‐1^+^ cells among CD4^+^ T cells after ZC3H13 deletion. Overall, these results indicate a close relationship between elevated CCND1 levels and an immunosuppressive post‐treatment TME characterized by exhausted CD4+ T‐cell features in HNSCC.

## 3 DISCUSSION

The limited efficacy of anti‐PD‐1 treatment in HNSCC was partly attributed to the emergence or enrichment of therapy‐adapted malignant states within the TME.^27^, ^28^ Previous single‐cell landscape studies of HNSCC have indicated that specific tumour‐associated clones and microenvironmental remodelling are central factors driving immunotherapy resistance.^10^, ^29^ Through single‐cell transcriptomics combined with multiplexed experimental models, this study identified a malignant tumour cell subpopulation characterized by high ZC3H13 expression that is significantly enriched after anti‐PD‐1 treatment and associated with poor therapeutic response. Our data demonstrate that ZC3H13 promotes intrinsic malignant phenotypes and is associated with exhaustion‐related transcriptional features in microenvironmental CD4^+^ T cells (Figure 7I).

ZC3H13 was first identified as a critical nuclear scaffold protein of the m6A methyltransferase complex (MTC), responsible for regulating the nuclear localization of the complex and maintaining the m6A methylation levels of transcripts.^30^, ^31^ However, the role of ZC3H13 in tumour biology appears to be highly tissue‐context dependent.^32^, ^33^ For instance, in colorectal cancer, ZC3H13 appears to function as a tumour suppressor, as its depletion facilitates tumour proliferation and invasion via Ras–ERK pathway activation.^34^ Conversely, our clinical cohort analysis, in vitro phenotypic assays, and the 4NQO‐induced conditional knockout mouse model yielded consistent conclusions: in HNSCC, particularly under immunotherapy pressure, our data support an oncogenic role of ZC3H13 and suggest its association with poor anti‐PD‐1 response. This discrepancy indicates that the biological functions of epigenetic regulators are strictly modulated by specific cell lineages and microenvironmental selection pressures.^35^, ^36^

Mechanistically, we identified CCND1 as a key downstream target of ZC3H13 through m6A‐seq. Previous studies have established that the regulatory fate of m6A‐modified target genes is highly dependent on the recruited reader proteins.^37^, ^38^ For example, YTHDF2 typically mediates the decay of target mRNAs, whereas IGF2BP family proteins (IGF2BP1/2/3) recognize m6A sites and envelope the target mRNAs, shielding them from ribonuclease attacks, thereby maintaining transcript stability and promoting translation.^22^, ^39^, ^40^ Our Actinomycin D tracking assays, mutant reporter gene assays, and RIP‐qPCR results provide direct evidence: ZC3H13‐regulated m6A modification of CCND1 mRNA favours IGF2BP1 association, thereby protecting CCND1 mRNA from degradation and maintaining its stability. It should be noted that ZC3H13 is unlikely to directly recognize *CCND1* mRNA in a sequence‐specific manner. Instead, based on these findings, ZC3H13 may function as a scaffold protein within the m6A methyltransferase complex and promote m6A modification of CCND1 mRNA. This modification subsequently facilitates the association of the m6A reader IGF2BP1 with *CCND1* mRNA, resulting in enhanced transcript stability. Therefore, the apparent specificity of the ZC3H13–CCND1 axis is likely mediated at least in part by m6A‐dependent reader recognition rather than direct RNA binding by ZC3H13 itself.

The core translational implication of this study is the identification of a mechanistic relationship between intrinsic tumour cell cycle regulation (CCND1) and immune‐suppressive microenvironmental features (CD4^+^ T cell exhaustion). As a core kinase regulatory subunit driving the G1/S transition, the overexpression of CCND1 not only accelerates solid tumour proliferation but has also been shown to profoundly affect the immune status of the TME.^41^, ^42^ Recent independent studies have demonstrated that inhibiting CDK4/6, the core downstream kinases of CCND1, can enhance effector T‐cell infiltration and activation while attenuating immunosuppressive checkpoint expression in tumour tissues.^43^, ^44^, ^45^ Our clinical sample analysis and single‐cell dimensionality reduction data (CytoTRACE) highly align with this theoretical framework: in the CCND1‐high TME, classic exhaustion markers of CD4^+^ T cells, such as PDCD1 (PD‐1), LAG3, and CTLA4, are significantly upregulated.^46^ This suggests that ZC3H13‐driven CCND1 accumulation may be linked to a less favourable immune contexture, although direct functional causality requires further investigation.

This may also explain why CCND1 expression was significantly associated with pathological grade but not clinical stage in our cohort. As a core cell‐cycle regulator, CCND1 may more directly reflect tumour cell proliferation, differentiation status, and histological aggressiveness, which are captured by pathological grade. In contrast, clinical stage is an anatomical and disease‐burden‐based parameter influenced by tumour size, local extension, nodal involvement, timing of diagnosis, and other anatomical or microenvironmental factors. Therefore, CCND1 expression may correlate with pathological grade without necessarily showing a parallel association with clinical stage. In addition, the limited cohort size and stage distribution may have contributed to the lack of statistical significance in the stage‐based comparison.

Based on these experimental results and theoretical foundations, we propose a mechanistic model for acquired immunotherapy resistance in HNSCC. Under the continuous selection pressure of anti‐PD‐1 therapy, malignant cells expressing high levels of ZC3H13 become enriched after treatment. The enriched ZC3H13 promotes m6A modification of *CCND1* mRNA, likely by facilitating the function of the m6A writer complex and recruits the reader IGF2BP1 to enhance its transcript stability. The substantial intracellular accumulation of CCND1 promotes the intrinsic malignant proliferation of the tumour and is accompanied by exhaustion‐related transcriptional features in microenvironmental CD4^+^ T cells, thereby constructing a solid immunosuppressive barrier. Our in vivo combination therapy experiments confirmed that epithelial ZC3H13 deletion reduces tumour burden and enhances anti‐PD‐1 responsiveness in the 4NQO‐induced model. Collectively, these findings support the role of the ZC3H13/IGF2BP1/CCND1 regulatory axis in HNSCC malignant progression and its association with poor anti‐PD‐1 response, suggesting that targeting this m6A epigenetic pathway can be seen as a promising clinical intervention method to reverse immunotherapy resistance in HNSCC.

## 4 METHODS

### 4.1 Clinical specimen collection

Ethical approval was obtained from the Ethics Committee of The First Affiliated Hospital of Sun Yat‐sen University (FAN‐SYSU; Approval No. [2025]855). The study procedures followed the Declaration of Helsinki, and each participant provided written informed consent. Formalin‐fixed paraffin‐embedded (FFPE) tumour specimens were collected from 120 patients with histologically confirmed HNSCC treated at FAN‐SYSU.

Of these patients, 90 received anti‐PD‐1‐based immunotherapy and had evaluable radiological responses according to the Response Evaluation Criteria in Solid Tumors (RECIST). Patients with complete or partial response (CR/PR) were defined as anti‐PD‐1 responders, and those with stable or progressive disease (SD/PD) as anti‐PD‐1 non‐responders.^47^ These patients were included in response‐specific subgroup analyses. The remaining 30 patients lacked evaluable anti‐PD‐1 response data and were therefore excluded from immunotherapy response analyses, but were retained for overall IHC expression, clinicopathological, and survival analyses where applicable.

### 4.2 Cell culture

The HOK cells were obtained through ScienCell Research Laboratories; SCC‐1 cells from Dr. T. Carey's laboratory; and human embryonic kidney 293T (HEK‐293T), SCC‐9, SCC‐15, and SCC‐25 cells through the American Type Culture Collection (ATCC); and UM1 cells from Dr. Xiaofeng Zhou at the University of Illinois at Chicago. Cells were grown at 37°C with 5% CO_2_ in DMEM/F12 medium containing 10% fetal bovine serum (FBS) and 1% penicillin‐streptomycin, detached with 0.25% trypsin‐EDTA at 80%–90% confluence, and used within 10 passages.

### 4.3 Plasmid construction and lentiviral transduction

Lentiviral shRNA constructs against ZC3H13 were generated using the pLKO.1 vector. Full‐length human ZC3H13 and CCND1 coding sequences were subcloned into pLV‐puro for overexpression. Recombinant plasmids were validated by DNA sequencing.

Lentivirus was produced in HEK‐293T cells. Briefly, cells at 70%–80% confluence were co‐transfected with the target plasmids and packaging plasmids with Lipofectamine 2000 (Invitrogen) as recommended by the manufacturer. After 48 h, lentiviral supernatants were harvested and filtered through a 0.45‐µm membrane.

HNSCC cells were lentivirally transduced with 8 µg/mL polybrene, followed by medium replacement after 24 h. Stable knockdown or overexpression cells were established by selection with 2 µg/mL puromycin for 7–14 days starting 48 h post‐infection. The shRNA target sequences and corresponding knockdown efficiencies are summarized in Table 1.

**TABLE 1:** shRNA target sequences used in this study.

Target Gene	Plasmid Name	Sequence (5' ‐ 3')	Knockdown efficiency
*ZC3H13*	sh_ZC3H13‐1	GCCACAGAACACGAGTAGAAA	SCC9: 77.5%; SCC25: 66.8%
*ZC3H13*	sh_ZC3H13‐2	GATTCTGACAATGGAGATATT	SCC9: 76.8%; SCC25: 63.3%
Control	Vector	TTCTCCGAACGTGTCACGT	–

### 4.4 RNA extraction and RT‐qPCR

Total RNA was extracted with TRIzol reagent (Invitrogen), and RNA concentration and purity were measured using a NanoDrop 2000 spectrophotometer (Thermo Fisher Scientific). complementary DNA (cDNA) was synthesized from 1 µg RNA using PrimeScript RT Master Mix (TaKaRa), followed by quantitative real‐time PCR (qPCR) with SYBR Green Premix Ex Taq II (TaKaRa) on a StepOnePlus system (Thermo Fisher Scientific). All assays included three biological replicates and technical triplicates. Relative ZC3H13 and CCND1 levels were quantified by the 2^−ΔΔCt^ method, with GAPDH served as the endogenous control. The primer sequences used for RT‐qPCR are listed in Table 2.

**TABLE 2:** Primer sequences used in this study.

Target Gene	Forward Primer Sequence (5' ‐ 3')	Reverse Primer Sequence (5' ‐ 3')
*ZC3H13*	TCTGATAGCACATCCCGAAGA	CAGCCAGTTACGGCACTGT
*CCND1*	GCTGCGAAGTGGAAACCATC	CCTCCTTCTGCACACATTTGAA
*GAPDH*	AGATCCCTCCAAAATCAAGTGG	GGCAGAGATGATGACCCTTTT

### 4.5 Western blot

HNSCC cells were lysed on ice in radioimmunoprecipitation assay (RIPA) buffer with protease/phosphatase inhibitors for 30 min, followed by centrifugation at 12 000 × g for 15 min at 4°C. Protein concentrations were measured using a BCA Protein Assay Kit. Proteins (20–30 µg) were separated by 10% SDS‐PAGE and transferred to PVDF membranes (Millipore). Membranes were blocked with 5% non‐fat milk for 1 h and incubated overnight at 4°C with antibodies against ZC3H13 (ab70802, Abcam, 1:1000), Cyclin D1 (26939‐1‐AP, Proteintech, 1:1000), YTHDC1 (29441‐1‐AP, Proteintech, 1:1000), YTHDF2 (24744‐1‐AP, Proteintech, 1:1000), IGF2BP1 (8482, Cell Signaling Technology, 1:1000), IGF2BP2 (14672, Cell Signaling Technology, 1:1000), and GAPDH (10494‐1‐AP, Proteintech, 1:5000), CDK1(DF6024, Affinity Biosciences, 1:1000), CCNB1(ab181593, Abcam, 1:2000), CCNA2(ab181591, Abcam, 1: 2000). After washing, membranes were incubated with horseradish peroxidase (HRP)‐conjugated secondary antibodies (SA00001‐2, Proteintech, 1:4000) for 1 h, followed by enhanced chemiluminescence (ECL) detection and ImageJ quantification.

### 4.6 RNA stability assay

mRNA stability was assessed using an actinomycin D‐mediated transcription inhibition assay. Stable ZC3H13‐knockdown, ZC3H13‐overexpressing, and control HNSCC cells were cultured in 6‐well plates to 70%–80% confluence, followed by treatment with actinomycin D (MCE, HY‐17559; 5 µg/mL). Cells were collected at 0, 6, and 12 h, and residual target mRNA levels were quantified by RT‐qPCR using TRIzol‐extracted RNA. mRNA levels at 0 h were normalized to 100%.

### 4.7 Dual‐Luciferase reporter assay

Wild‐type (WT) or mutant (Mut) CCND1 sequences containing predicted m6A sites were cloned into the pmirGLO luciferase reporter vector and co‐transfected into HEK‐293T cells with ZC3H13 knockdown or overexpression plasmids. At 48 h after transfection, reporter activity was quantified with the MedChemExpress dual‐luciferase kit (HY‐K1013), and Firefly luciferase values were normalized to Renilla luciferase to assess the effect of ZC3H13 on CCND1 regulation.

### 4.8 RNA Immunoprecipitation

HNSCC cell lysates were prepared with protease inhibitor‐containing immunoprecipitation (IP) lysis buffer (Beyotime, P0013). To eliminate genomic DNA contamination, the lysates were subjected to DNase treatment prior to centrifugation. For RIP‐qPCR, 50 µL clarified lysate was reserved for input control, and the remainder was incubated overnight at 4°C with 2 µg of anti‐YTHDC1 (29441‐1‐AP, Proteintech), anti‐YTHDF2 (24744‐1‐AP, Proteintech), anti‐IGF2BP1 (8482, Cell Signaling Technology), anti‐IGF2BP2 (14672, Cell Signaling Technology), or control immunoglobulin G (IgG). Complexes were captured using Pierce Protein A/G Magnetic Beads (Thermo Fisher Scientific, 88803) for 4 h at 4°C. Following 3 stringent washes, RNA from the immunoprecipitates was purified and analysed by RT‐qPCR to determine the enrichment of target transcripts.

### 4.9 Cell proliferation assay

Cell growth was evaluated with the CCK‐8 assay (Dojindo). HNSCC cells were plated at 2 × 10^3^ cells/well in 96‐well plates. At 0, 24, 48, 72 and 96 h, CCK‐8 reagent (10 µL/well) was added, and cells were incubated at 37°C for 2 h. Cell growth curves were generated from optical density (OD)450 values.

### 4.10 Colony formation assay

Stably transduced cells were plated in 6‐well plates at 500–1000 cells/well and cultured for 14 days at 37°C with 5% CO_2_. Colonies were washed with phosphate‐buffered saline (PBS), fixed for 20 min, stained with 0.1% crystal violet for 30 min, photographed, and counted if they contained ≥50 cells.

### 4.11 Transwell migration and invasion assays

Migratory and invasive capacities were examined using 8‐µm Transwell inserts (Corning), with Matrigel‐coated chambers (BD Biosciences) used for invasion assays. A total of 5 × 10^4^ cells in serum‐free medium were loaded into the upper chamber, and 20% FBS‐containing medium was added to the lower chamber. After 24–48 h, migrated or invaded cells were fixed, crystal violet‐stained, and quantified in five random fields.

### 4.12 Single‐cell RNA sequencing analysis

The scRNA‐seq dataset was retrieved from NCBI Gene Expression Omnibus (GEO) database using accession GSE195832. The dataset was derived from a neoadjuvant anti‐PD‐1 trial in advanced HNSCC patients treated with nivolumab and included paired pre‐ and post‐treatment tumour samples from four patients (Pt1–Pt4). Data were processed in R v4.2.0. Low‐quality cells were filtered out if they contained < 200 or > 6000 detected genes or > 10% mitochondrial gene content. After normalization, batch‐effect removal with harmony, principal component analysis (PCA) was applied to reduce dimensionality, whereas uniform manifold approximation and projection (UMAP) was used to visualize cell clustering patterns.

To define malignant tumour cell subclusters, CopyKAT‐confirmed aneuploid epithelial cells were extracted and re‐clustered using Seurat. The post‐treatment‐enriched malignant cluster was defined according to the following criteria: (i) malignant identity confirmed by CopyKAT‐inferred aneuploidy; (ii) assignment as a distinct tumour cell subcluster by unsupervised clustering; and (iii) enrichment in post‐treatment samples based on the proportional distribution of malignant cells across paired pre‐ and post‐treatment samples. Subsequently, the CytoTRACE framework was performed to assess the differentiation potential of malignant cells; higher scores indicate a more primitive and stem‐like cellular state. Finally, T‐cell exhaustion scores were determined via the AddModuleScore function in Seurat. The exhaustion‐related gene set included *PDCD1, CTLA4, LAG3, IL2, TNF* and *TNFRSF18*. Scores were calculated within annotated T‐cell subsets, particularly CD4+ T cells, and were compared across pre‐treatment and post‐treatment samples.

### 4.13 m6A methylated RNA immunoprecipitation sequencing (MeRIP‐seq)

MeRIP‐seq was used to profile m6A methylation in HNSCC cells. Purified mRNA was fragmented into ∼100‐nt fragments, with part retained as input and the remainder immunoprecipitated using magnetic bead‐conjugated m6A antibody (Synaptic Systems, 202003; 2 µg) at 4°C for 12 h. m6A‐enriched RNA was then washed, eluted, and recovered.

Sequencing libraries were constructed from both immunoprecipitated RNA and input samples, followed by paired‐end sequencing. Following quality control and genome alignment, m6A peaks were called using exomePeak2 with thresholds of false discovery rate (FDR) < 0.05 and fold enrichment > 2. Motif enrichment analysis was performed on the identified peak regions using HOMER software.

### 4.14 Animal models

K14CreER; ZC3H13^fl/fl mice^ were established by crossing ZC3H13^fl/fl mice^ from Cyagen Biosciences with K14CreER mice from The Jackson Laboratory to achieve epithelial‐specific ZC3H13 deletion in the oral mucosa. Mice were housed under specific pathogen‐free (SPF) conditions with a 12‐h light/dark cycle and ad libitum access to food and water.

For Cre induction, tamoxifen (Sigma, T5648) was prepared at 20 mg/mL in 10% ethanol/corn oil using corn oil (Aladdin, C116025) as the vehicle. Mice received intraperitoneal injections of 100 µL tamoxifen solution once daily for 3 consecutive days before 4NQO exposure.

For establishment of the chemically induced oral carcinogenesis model, 6–8‐week‐old mice received 4NQO (Sigma, N8141) in drinking water for 16 weeks at 50 µg/mL before switching to regular drinking water until week 26. For PD‐1 blockade, mice received anti‐PD‐1 antibody (BE0146, BioXcell, Shanghai, China) or isotype‐matched IgG beginning at week 22. Antibodies were administered intraperitoneally at 200 µg per mouse on treatment Days 1, 3, 5, 7 and 14. Mice were euthanized at Week 26 for endpoint analyses.

Body weight and oral lesion development were monitored weekly throughout the experiment. At the endpoint, tongue and oral mucosal tissues were collected and fixed in 4% paraformaldehyde before H&E and IHC staining to assess histopathological changes and target protein expression in 4NQO‐induced oral tumorigenesis.

### 4.15 H&E and IHC staining

Oral tissues from 4NQO‐induced mice and HNSCC specimens were analysed by H&E and IHC staining. Tissues were fixed for 24 h and processed into 5‐µm paraffin sections. H&E sections were assessed by pathologists for epithelial dysplasia and squamous cell carcinoma invasion.

For IHC, after deparaffinization and rehydration, microwave antigen retrieval was performed in EDTA buffer (pH 9.0), sections were treated with 3% H_2_O_2_, blocked with 5% bovine serum albumin (BSA), and incubated overnight at 4°C with the indicated primary antibodies.: ZC3H13 (ab70802, Abcam, 1:200), Cyclin D1 (26939‐1‐AP, Proteintech, 1:200), anti‐Ki67 (NB500‐170, Novus, 1:200), and anti‐PCK (pan‐cytokeratin, sc‐8018, Santa Cruz, 1:200). After HRP‐conjugated secondary antibody incubation, signals were developed using 3,3′‐diaminobenzidine (DAB), followed by haematoxylin counterstaining. IHC staining was semi‐quantified using the histochemical score (H‐score) (0–300), and patients were classified as high or low expressers based on the median H‐score for each marker.

### 4.16 Multi‐colour immunofluorescence staining and quantitative image analysis

FFPE tissues were sectioned at 4 µm, deparaffinized, and incubated with 0.3% H_2_O_2_/methanol for 10 min. Antigens were retrieved in sodium citrate buffer at 95°C for 5 min, followed by washes with running water and 1× Tris‐buffered saline with Tween‐20 (TBST) and blocking for 10 min.

Multiplex immunofluorescence staining was performed sequentially, with one primary antibody applied in each cycle. Sections were incubated with the indicated primary antibodies at room temperature for 1 h: CD4 (HY‐P83756, MCE, 1:500), CCND1 (HY‐P80098, MCE, 1:50), and PD‐1 (84651, Cell Signaling Technology, 1:100) in the optimized order of CD4, CCND1, and PD‐1. Following three 5‐min washes with 1× TBST, sections were treated sequentially with polymer HRP‐conjugated anti‐mouse/rabbit IgG secondary antibody and tyramide signal amplification (TSA) fluorophore reagent (TissueGnostics, TGT5C100; 1:100) for 10 min each. Two 3‐min washes with 1× TBST were then performed. Prior to each subsequent staining round, microwave‐mediated antigen retrieval was repeated in sodium citrate buffer (pH 6.0). After all target antigens had been labelled, nuclei were counterstained with DAPI (Roche, 10236276001). Negative control sections were treated in parallel by omitting the primary antibody.

Multiplex immunofluorescence images were acquired from regions of interest using the TissueFAXS platform (TissueGnostics). Images were recorded in four fluorescence channels corresponding to fluorescein isothiocyanate (FITC), Cy3, Cy5, DAPI. Cells with the indicated phenotypes were quantified using TissueQuest software, with positivity thresholds defined according to positive controls. Cell‐to‐cell contact analysis was performed using StrataQuest software (TissueGnostics), and a minimum overlap of 3 pixels between adjacent cellular signals was required to define a contact event, as previously described.

### 4.17 Polysome profiling

To assess whether ZC3H13 depletion alters global translational activity, polysome profiling was carried out. HNSCC cells were exposed to cycloheximide (100 µg/mL) to arrest ribosome elongation and subsequently lysed on ice. Clarified lysates were separated by ultracentrifugation on 10%–50% linear sucrose gradients. Ribosome profiles were generated by collecting fractions with a gradient fractionator while recording absorbance at 254 nm.

### 4.18 Statistical analysis

Statistical processing was conducted with GraphPad Prism 8.0 and R v4.2.0. Quantitative results are reported as mean ± standard deviation from no fewer than three independent biological replicates. Student's *t*‐test was applied for two‐group comparisons, and one‐way analysis of variance (ANOVA) with Tukey's post hoc test for comparisons among multiple groups. Pearson correlation was used to examine relationships between gene expression levels. Kaplan–Meier analysis and Cox proportional hazards modelling were used to examine survival outcomes. Statistical significance was defined using a two‐sided threshold of *p *< .05.

## AUTHOR CONTRIBUTIONS

W.Q.C., Y.L., S.C. and C.H.Z. performed the experiments and analysed the data. Z.L., K.S. and C.L.Y. assisted with methodology, data collection and data interpretation. K.L. and W.L. conceived and supervised the study. W.Q.C. drafted the manuscript. All authors critically revised the manuscript, approved the final version, and agree to be accountable for all aspects of the work.

## CONFLICT OF INTEREST STATEMENT

The authors declare no conflicts of interest.

## ETHICAL APPROVAL

The study was approved by the Ethics Committee of the First Affiliated Hospital of Sun Yat‐sen University (Approval No. [2025]855) and conducted in accordance with the Declaration of Helsinki. Written informed consent was obtained from all participants prior to specimen collection.

## Supporting information



Supporting Information

Supporting Information

## Data Availability

The public single‐cell RNA‐seq dataset analysed in this study was obtained from the Gene Expression Omnibus (GEO) under accession number GSE195832. Public bulk RNA‐seq and clinical data for HNSCC were obtained from The Cancer Genome Atlas through the Genomic Data Commons (GDC) Data Portal under project ID TCGA‐HNSC. Additional data supporting the findings of this study are available from the corresponding authors upon reasonable request.
