# Supplementary material for: ZC3H13‐mediated m6A stabilization of CCND1 promotes malignant progression and is associated with poor anti‐PD‐1 response in HNSCC
Source: Clin Transl Med. 2026 Jul 27;16(8):e70750. doi: 10.1002/ctm2.70750 (PMC13403043; doi:10.1002/ctm2.70750)
Supplement: Supplementary file 1 — Supporting Information [file CTM2-16-e70750-s001.docx]

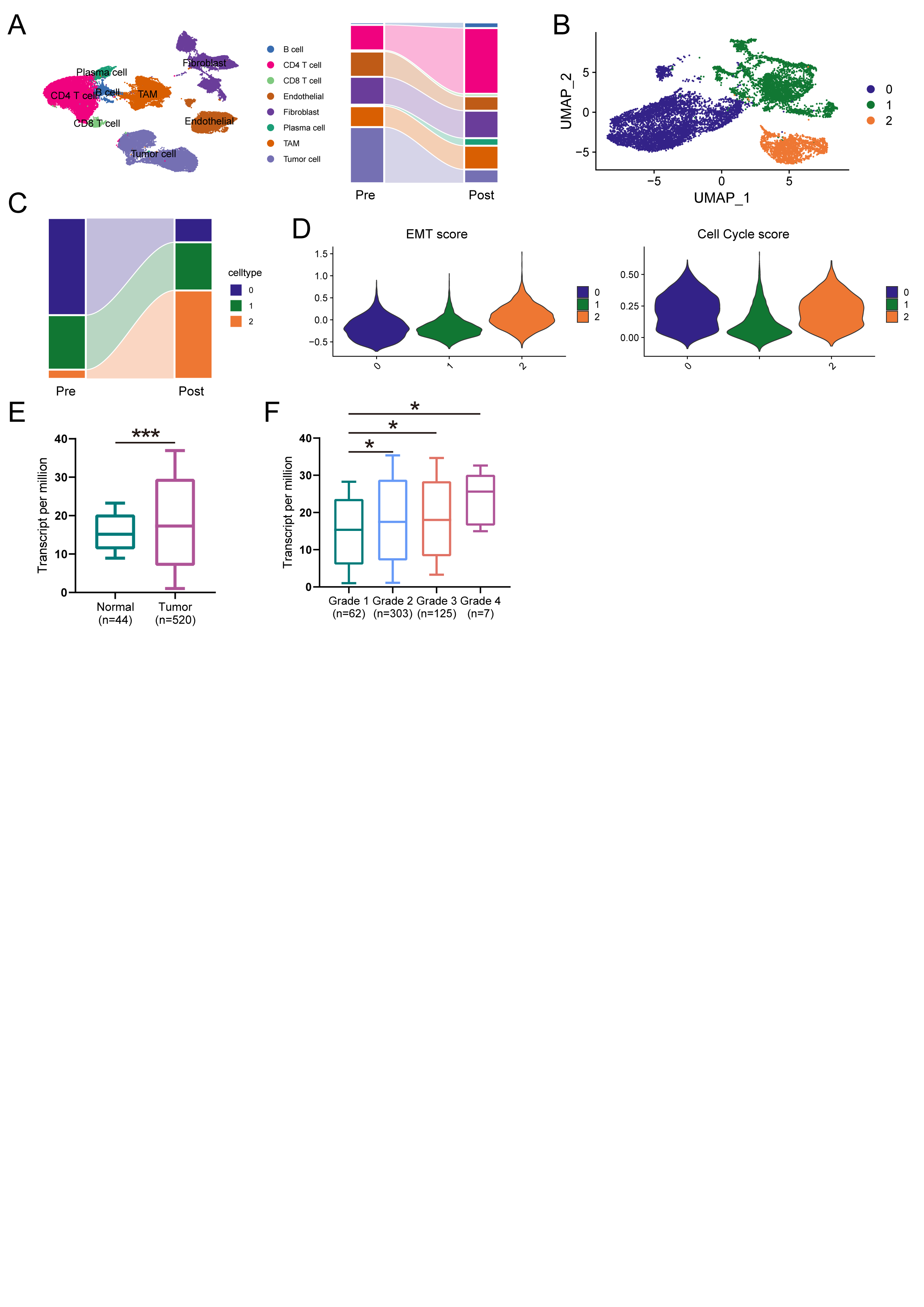


Supplementary Figure 1. Single-cell immune landscape of HNSCC and validation in the TCGA cohort.

1. Left: UMAP plot identifying the major cell types in HNSCC (e.g., T cells, B cells, tumor cells, fibroblasts); Right: Alluvial plot showing the shift in cell type proportions before and after treatment.
2. UMAP clustering visualization of the three primary malignant cell sub-populations (Clusters 0, 1, and 2).
3. Alluvial plot illustrating the proportional distribution of malignant sub-populations in Pre- and Post-treatment samples.
4. Violin plots assessing the EMT scores and Cell Cycle scores across different malignant sub-populations.
5. Analysis of ZC3H13 transcript levels between normal (n=44) and tumor (n=520) tissues based on the TCGA HNSCC cohort.

(F) Statistical trend of ZC3H13 expression levels across different pathological grades (Grade 1–4) in the TCGA cohort.
